# Bovine Hemoglobin Enzymatic Hydrolysis by a New Eco-Efficient Process-Part II: Production of Bioactive Peptides

**DOI:** 10.3390/membranes10100268

**Published:** 2020-09-29

**Authors:** Mira Abou-Diab, Jacinthe Thibodeau, Barbara Deracinois, Christophe Flahaut, Ismail Fliss, Pascal Dhulster, Laurent Bazinet, Naima Nedjar

**Affiliations:** 1 Department of Food Science, Université Laval, Québec, QC G1V 0A6, Canada; mira.abou-diab.1@ulaval.ca (M.A.-D.); jacinthe.thibodeau.1@ulaval.ca (J.T.); ismail.fliss@fsaa.ulaval.ca (I.F.); 2 Laboratory of Food Processing and Electromembrane Process (LTAPEM), Université Laval, Québec, QC G1V 0A6, Canada; 3 Institute of Nutrition and Functional Foods (INAF), Université Laval, Québec, QC G1V 0A6, Canada; 4 UMR Transfrontalière BioEcoAgro N°1158, Université Lille, INRAE, Université Liège, UPJV, YNCREA, Université Artois, Université Littoral Côte d’Opale, ICV—Institut Charles Viollette, F-59000 Lille, France; barbara.deracinois@univ-lille.fr (B.D.); christophe.flahaut@univ-artois.fr (C.F.); pascal.dhulster@univ-lille.fr (P.D.)

**Keywords:** bovine hemoglobin, electrodialysis with bipolar membrane, bioactive peptides, antibacterial activity, antifungal activity, antioxidant activity

## Abstract

Bovine cruor, a slaughterhouse waste, was mainly composed of hemoglobin, a protein rich in antibacterial and antioxidant peptides after its hydrolysis. In the current context of food safety, such bioactive peptides derived from enzymatic hydrolysis of hemoglobin represent potential promising preservatives for the food sector. In this work, the hemoglobin hydrolysis to produce bioactive peptides was performed in a regulated pH medium without the use of chemical solvents and by an eco-efficient process: electrodialysis with bipolar membrane (EDBM). Bipolar/monopolar (anionic or cationic) configuration using the H^+^ and OH^−^ generated by the bipolar membranes to regulate the pH was investigated. The aim of this study was to present and identify the bioactive peptides produced by EDBM in comparison with conventional hydrolysis and to identify their biological activity. The use of the EDBM for the enzymatic hydrolysis of hemoglobin has allowed for the production and identification of 17 bioactive peptides. Hydrolysates obtained by EDBM showed an excellent antimicrobial activity against six strains, antioxidant activity measured by four different tests and for the first time anti-fungal activities against five yeasts and mold strains. Consequently, this enzymatic hydrolysis carried out in regulated pH medium with bipolar membranes could provide bioactive peptides presenting antibacterial, antifungal and antioxidant interest.

## 1. Introduction

According to a report published in 2018 by The World Bank, by 2050, the world is expected to generate 3.40 billion tons of waste annually, increasing drastically from today’s 2.01 billion tons [1]. Hence, the industrial processes used in the food industries participate to this growing production of waste driven by rapid urbanization and growing populations. To move towards a circular or sustainable economy, the valorization of the many bio food industry co-products, underestimated and underused, by producing bioproducts with high added value, becomes a key issue. The circular economy is an economy operating in a loop and thus avoiding waste. Its objective is to produce goods but at the same time to strongly limit the consumption of non-renewable energy sources and raw materials.

Amongst these agri-food wastes, bovine blood from slaughterhouses, is a natural bioresource produced in all countries around the world. Billions of tons of blood are collected every year, and they are processed into blood meal and sold as low-value animal food and fertilizer or discarded as effluent [2,3]. This large quantity of animal blood generated is a problematic by-product for the meat industry since it has a very high pollutant load when discarded into the environment with minimal treatments [4,5]. The Cruor represents 40% of the whole blood and contains mainly hemoglobin (90%), a protein broadly described as a rich source of bioactive peptides after its enzymatic hydrolysis [6,7,8]. Various biological activities, including opioid activity [9,10,11], hematopoietic [12] and antihypertensive [13] have been reported for hemoglobin protein hydrolysate. The most described activities are antimicrobial [14,15,16] and antioxidant activities [17]. Many studies have shown that bioactive peptides derived from conventional enzymatic hydrolysis of hemoglobin can represent a natural alternative to synthetic preservatives that are suspected to induce pathological and toxic effects [18,19,20]. These peptides have been used recently to protect meat, reducing lipid oxidation and microbial growths during food storage and distribution [17]. Hence, in link with the concept of the circular economy, blood from slaughterhouses can thus be recycled by the meat industry for the production of biopeptides with improved functions. Furthermore, this is also in direct link with the current context of food safety and food protection by means of natural products, adding value to proteins from wastes [5,21,22].

However, the conventional process of hydrolysis includes several steps consisting of hemoglobin denaturation by chemical acidification, hydrolysis with pepsin enzyme and pH adjustment during hydrolysis. Due to the use of chemical basis and acids, the final hydrolysates contain high levels of mineral salts. According to recent life cycle assessments, an electro-membrane process recognized as green process [23,24,25] electrodialysis with a bipolar membrane (EDBM) was tested with two different membrane configurations (cationic (MCP) and anionic (AEM) membranes) as described by Abou-Diab et al., 2020 (part 1) [26]. It was reported that hydrolysis in EDBM showed the same enzymatic mechanism «Zipper» as observed in conventional hydrolysis. Moreover, EDBM allowed the generation of α137-141 in the same concentration as for the conventional hydrolysis. EDBM-MCP allowed the production of hydrolysates containing a low concentration of mineral salts while EDBM-AEM, allows the production of hydrolysates with a similar salt content as conventional hydrolysis. This was due to the specific characteristics of the bipolar membranes which allow to generate in situ H^+^ ions from water dissociation under an electrical field and consequently no need to use any chemical agent during hydrolysis. However, this study focused on the production of α137-141 peptide and did not test the bioactivities of these hydrolysates produced by this new and eco-efficient technology.

The aim of the present study was to produce bioactive peptides derived from enzymatic hydrolysis of bovine hemoglobin using EDBM. The objectives of this study were to (1) identify and characterize the peptide population of the different conditions by UPLC-MS/MS, (2) identify the bioactive peptides resulting from this hydrolysis of hemoglobin and (3) study the biological activities of the hydrolysates obtained from EDBM. First, the antibacterial potential was investigated using the agar diffusion method and minimum inhibitory concentration against six bacteria. Next, the antifungal activity was evaluated using the agar diffusion method against five strains. At the end, the antioxidant potential was investigated using several measurements, including β-carotene bleaching inhibition activity, DPPH radical scavenging activity assay, ABTS radical scavenging activity assay and evaluation of total antioxidant capacity to provide a clear idea about their real antioxidant potential.

## 2. Materials and Methods

### 2.1. Materials

Reagents. KCl was obtained from EMD Chemicals Inc., USA, Na_2_SO_4_ was supplied by ACP Inc. (Montréal, QC, Canada), KOH was purchased from Fisher Scientific (New Jersey, NJ, USA) and HCl was purchased from Anachemia, VWR Company International (ON, Canada). Chemicals required for the assays including 1,1-diphenyl-2-picrylhydrazyl (DPPH), Butylated hydroxytoluene (BHT), 2,2′-azino-bis 3-ethylbenzothiazoline-6-sulphonic acid (ABTS), 6-hydroxy-2,5,7,8-tetramethylchroman-2-carboxylic acid (Trolox), β-carotene, and linoleic acid were purchased from Sigma-Aldrich (Saint-Quentin Fallavier, France). Neokyotorphin (α137-141) was purchased from GeneCust (Boynes, France). The ultrapure water was prepared using a Milli-Q system.

Cultures. *Listeria monocytogenes* (ATCC 19112), *Staphylococcus aureus* (ATCC 13709), *Micrococcus luteus* (ATCC 9341), *Escherichia coli* (ATCC 8733) and *Salmonella Newport* (ATCC 6962) were from the American Type Culture Collection (ATCC, Rockville, MD, USA). *Kocuria rhizophila* (CIP 53.45), was from Collection de l’Institut Pasteur (CIP, Paris, France). *Aspergillus Niger* (3071-13) and *Paecilomyces spp* (5332-9a) were isolated from dairy product (strain collection of Denis Roy, Laval University, Québec, QC, Canada)*, Mucor racemosus* (LMA-722) was obtained from Laboratoire de mycologie alimentaire (Laval university, Québec, QC, Canada)*, Penicillium crustosum* (27,159) and *Rhodotorula mucilaginosa* (27,173) were isolated from dairy product (strain collection of General Mills Yoplait, France).

Pepsin. The pepsin lyophilized powder from porcine gastric mucosa was purchased from Sigma-Aldrich (P6887, Oakville, ON, Canada). It was used for the production of the hydrolysate. Pepsin activity was determined according to a protocol established by the supplier Sigma-Aldrich (Saint-Quentin Fallavier, France). Pepsin was stored at −20 °C.

Hemoglobin. The purified hemoglobin powder from bovine blood, dark brown, was purchased from Sigma-Aldrich (H2625, Oakville, ON, Canada). Hemoglobin was stored at 4 °C before uses.

### 2.2. Preparation of Bovine Hemoglobin Hydrolysates

As described by Abou-Diab et al., 2020 [26], three conditions have been set up: hydrolysis by electrodialysis with bipolar and cationic membranes «EDBM-MCP», hydrolysis by electrodialysis with bipolar and anionic membranes «EDBM-AEM» and conventional hydrolysis in beaker considered as «control».

#### 2.2.1. Stock Solution Preparation

In total, 15 g of purified bovine hemoglobin (BH) was solubilized in 100 mL of ultrapure water. After that, the solution was centrifuged at 6000× *g* for 30 min (Eppendorf AG, 22331 Hamburg, Centrifuge 5804 R, Brinkmann Instruments, Westbury, NY, USA). Drabkin’s method was later done on the recovered supernatant to determine the real BH concentration (C_BH_) [27]. From the concentration C of the stock solution, a solution of purified BH was prepared by dilution to a 1% (*w/v*) precise concentrations of hemoglobin C_BH_.

#### 2.2.2. Hydrolysis Process

Briefly, and according to Abou-Diab et al., 2020 (part 1) [26], hydrolysis was carried out at pH 3 to denature the hemoglobin solution, at a constant temperature of 30 °C. pH was adjusted to 3 with hydrochloric acid 2 M in conventional hydrolysis and adjusted to 3 by bipolar membrane which generated the H^+^ ions from water dissociation under an electrical field in EDBM-MCP and EDBM-AEM. The hydrolysis reaction was started by the addition of pepsin (EC 3.4.23.1, 3200–4500 units mg^−1^ protein) at a 1:11 (mole/mole) enzyme/protein ratio. Samples were collected at T_0_, T_2.5_, T_30_, T_60_, T_120_ and T_180_ min of hydrolysis, corresponding to different degrees of hydrolysis. In total, 0.5 M of potassium hydroxide was added to stop the peptic hydrolysis reaction by adjusting the pH to 9. The samples (triplicates) were stored at −20 °C, and then they were lyophilized, and the recovered powder was ready for testing. The current intensity, conductivities, pH and temperature, for both 1% HB and KCl solutions, were recorded every 30 min during EDBM treatments.

### 2.3. Electrodialysis Cell

**Figure 1:**
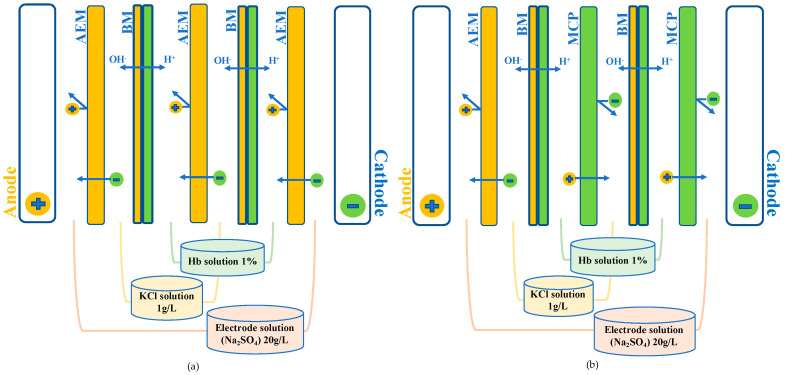
Schematic representation of electrodialysis cell configurations used for electro-acidification and hydrolysis of bovine hemoglobin in (**a**) electrodialysis with bipolar and anion exchange membranes (EDBM-AEM) and (**b**) electrodialysis with bipolar and monovalent cation permselective membranes (EDBM-MCP). MCP: monovalent cation permselective membrane; AEM: anion exchange membrane; BM: bipolar membrane.

The electrodialysis (ED) cell was a Microflow-type cell with 10 cm^2^ of effective membrane surface, purchased from ElectroCell AB (Taby, Sweden). The anode used was a dimensionally stable electrode and the cathode was a 316 SS electrode. A BK Precision power supply (Model BK9110-ND, Vancouver, BC, Canada) was employed at a constant voltage of 15 V, corresponding at the beginning of the process to a current density of 5.3 mA/cm^2^. The cell configurations are those presented previously by Abou-Diab et al. [26] and displayed in Figure 1. Two ED configurations were tested: the ED configuration named «EDBM-AEM» (Figure 1a) formed by stacking three anion-exchange membranes (AEM, Astom, Tokyo, Japan) and two bipolar membrane (BM, Astom, Tokyo, Japan) and the ED configuration named «EDBM-MCP» (Figure 1b) stacking one anion-exchange membrane (AEM, Astom, Tokyo, Japan), two monovalent cation permselective membranes (MCP, Japan Food grade Neosepta, Astom, Tokyo, Japan) and two bipolar membranes (BM, Astom, Tokyo, Japan). In both cases, the ED configuration defined three closed loops containing 200 mL of hemoglobin solution (1% Hg *w/v*), 200 mL of salt ion recovery (KCl 1 g/L (EMD Chemicals Inc, Port Wentworth, GA, USA)), and 250 mL of electrode-rinsing solution (Na_2_SO_4_ 20 g/L (ACP Inc., Montréal, QC, Canada)).

From the results of the technological part of this study (Abou-Diab et al., 2020 (part 1) [26]), EDBM, in batch mode by recirculation of the solution until pH 3 was obtained, was efficient to denature hemoglobin. The mean times required to electro-acidify the 1% HB solution were 390 ± 20 min and 60 ± 2 min in EDBM-MCP and EDBM-AEM, respectively, while the global process durations to produce peptides (electro-acidification + hydrolysis) were 570 ± 20 min and 240 ± 2 min in EDBM-MCP and EDBM-AEM, respectively. Conductivity values tend to decrease for cationic configuration because of partial demineralization (34%) caused by EDBM-MCP configuration and tend to increase for anionic configuration due to partial mineralization (88%) caused by migration of anions from KCl solution. Furthermore, EDBM-MCP, allowed the production of α137-141 peptide with a low mineral content but the MCPs exhibited a fouling, due to electrostatic interactions between peptides (positively charged) and the membranes (negatively charged). In comparison, EDBM-AEM, allowed for the production of the α137-141 peptide more rapidly without membrane fouling. The subsequent sections will focus on identifying the biological activity of the hydrolysates produced.

### 2.4. Analysis

#### 2.4.1. RP-UPLC

##### Materials, Software and Elution Program Used

All samples were analyzed with two different UPLC to identify peptide population and bioactive peptides.

First method: RP-UPLC analyses performed on all samples used a 1290 Infinity II UPLC (Agilent Technologies, Santa Clara, CA, USA). The RP-UPLC apparatus was composed of a binary pump (G7120A), a multisampler (G7167B), an in-line degasser and a variable wavelength detector (VWD G7114B) adjusted to 214 nm. 0.22 µm PVDF filter were used to filter hydrolysis fractions into a glass vial. Samples were loaded (0.25 µL) into a Poroshell 120 EC-C18 column (2.1 × 100 mm i.d., 2.7 micron, Agilent, Santa Clara, CA, USA). A flow rate of 500 µL/min at 23 °C was used to operate the column. A gradient was applied for the mixture of solvent A (LC-MS grade water with 0.1% formic acid) and solvent B (LC-MS grade ACN with 0.1% formic acid) with solvent B increasing from 1% to 13% in 6 min, to 35% until 25 min, and to 100% until 35 min and holding until 45 min, then back to initial conditions. Each sample analysis was performed in triplicate for ensuring technical reproducibility.

Second method: After sample drying, peptides were dissolved in H2O/0.1% TFA at 0.5 mg/mL and centrifuged for 5 min at 10,000× *g*. 10 µL were separated by chromatography at 30 °C on an ACQUITY UPLC system (Waters Corporation, Guyancourt, France) equipped with a Kinetex 2.6 µm C18 100 Å (150 × 4.6 mm) column (Phenomenex). Acetonitrile gradient was set at 0.5 mL/min (the mobile phases were composed of solvent A (0.1% (*v/v*) formic acid/99.9% (*v/v*) water) and solvent B (0.1% (*v/v*) formic acid/99.9% (*v/v*) acetonitrile): from 5% to 15% solvent B during the first 30 min, from 15% to 30% over 60 min, from 30% to 50% over 10 min and then maintained during 10 min at 95% solvent B. The eluate was then directed into the electrospray ionization source of the qTOF Synapt G2-Si™ (Waters Corporation). The analysis was performed in sensitivity, positive ion and data dependent analysis (DDA) modes. The source temperature was at 150 °C and the capillary and cone voltages were fixed, respectively, at 3000 and 60 V. Data for *m/z* values were collected in the range of 100 to 2000 Da with a scan time of 0.2 s. A maximum of 10 precursor ions were chosen for MS/MS analysis with an intensity threshold of 100,000 with A Collision-induced dissociation (CID) fragmentation mode and a scan time of 0.1 s were chosen for MS/MS data collection.

##### Identification of Peptide Population

PEAKS Studio (version 8.5, Bioinformatics Solutions Inc., Waterloo, ON, Canada) software and its database search tools were used for peptide identification. The UniProt database restricted to Bos Taurus was used. A mass tolerance of 35 ppm, 3 missing cleavage sites and an MS/MS tolerance of 0.2 Da were allowed while a variable methionine oxidation was considered. Protein and peptide identity relevance was judged according to their score in the research software (*p* value of 0.05 (*p* < 0.05), False Discovery Rate < 1%). For the search of peptides from the pepsic hydrolysate by Peaks^®^ software, the Bos taurus bovine hemoglobin alpha and beta chain sequences were taken as model proteins. The number of unique and common peptides identified in all samples were subsequently presented in Venn diagram format using R software (R Core Team (2013) [28].

##### Identification of Bioactive Peptides by Mass Spectrometry Analyses

To identify and quantify the relative abundances of the peptides present in the samples, a hybrid ion mobility quadrupole time of flight mass spectrometer 6560, IM-Q-TOF, Agilent, Santa Clara, USA) was used. All LC-MS/MS experiments were acquired using Q-TOF. A positive mode at Extended Dynamic Range, 2 Ghz, 3200 *m/z* with a scan range between 100 and 3200 *m/z* was used to record the MS/MS signals. The drying gas was nitrogen (purity 99,999% UHP-T (Praxair, Québec, QC, Canada)) at 13.0 L/min and 150 °C and was also used as nebulizer gas at 30 psig. The capillary voltage was set at 3500 V while the nozzle voltage at 300 V and the one of the fragmentor at 400 V. The instrument calibration was carried out by using an ESI-L low concentration tuning mix (G1969-85000, Agilent Technologies, Santa Clara, CA, USA). Data acquisition and analyses were performed with the Agilent Mass Hunter Software package (LC/MS Data Acquisition, Version B.08.00 and Qualitative Analysis for IM-MS, Version B.07.00 Service Pack 2 with BioConfirm Software, Santa Clara, CA, USA).

UPLC-MS/MS coupling was used to analyze the peptides. This coupling, offering the advantage of a complementarity between UV profile from the UPLC and the masses of the corresponding compounds for each point of the chromatogram. This also gives the amino acid composition of each compound. Several parameters were considered to ensure a good identification of bioactive peptides: the retention time, the monoisotopic molecular mass and the second-order derivative spectral analysis of peptides testified of the presence (or absence) of aromatic amino acids [29]. The presence of tyrosine (Tyr), tryptophan (Trp) or phenylalanine (Phe) in the peptide sequence indicates the presence of bioactive peptide. The peptides have been identified by comparing them with the known hemoglobin alpha and beta sequences with the BioConfirm software. Then bioactive peptides were identified by comparing them to the literature [8,9,13,30,31,32].

#### 2.4.2. Biological Activities

##### Antibacterial Activity

- Agar diffusion method

The antibacterial activity was determined by the method of Yaba Adje [33]. Six bacterial species were tested for determination of the antibacterial activity: *Staphylococcus aureus* (ATCC 13709), *Listeria monocytogenes* (ATCC 19112), *Micrococcus luteus* (ATCC 9341), *Kocuria rhizophila* (CIP 53.45), *Escherichia coli* (ATCC 8733) and *Salmonelle Newport* (ATCC 6962). These bacterial species are commonly responsible for food alteration [34,35]. These bacterial species were stored at −20 °C in glycerol containing nutrient broth and were sub-cultured twice in Muller–Hinton broth (MH) at 37 °C under agitation (60 rpm). After 24 h of incubation at 37 °C in nutrient broth, absorbance of the pre-cultures was determined at 620 nm using the medium MH as a blank. A dilution series of 1/100 and of 1/1000 were carried out in tryptone salt (TS) for gram (+) and gram (−) bacteria, respectively, in order to adjust inoculum concentration to ×10^6^ CFU/mL. Seeding was carried out by the flooding method on petri dishes containing 15 mL of MH agar previously casted and dried. A volume of 10 μL of the samples filtered at 0.25 μm, as well as the positive control, were then deposited on the surface of the agar in the form of a spot and plates were incubated 24 h at 37 °C. The antibacterial activity was measured as the diameter of the clear zone of growth inhibition compared with a positive control, Ampicillin (0.1 mg/mL) for all bacterial species and Colistin (0.1 mg/mL) for *Escherichia coli*. This antimicrobial test was performed in triplicate.

- Minimal inhibitory concentration (MIC) determination

The MIC (Minimum Inhibitory Concentration) corresponds to the lowest concentration of the peptide hydrolysates allowing the inhibition of the bacterial strain growth. It is a liquid growth inhibition assay performed in a sterile 96-well microplate, of which the response was read after 24 h incubation [36]. This test makes it possible to quantify the antimicrobial activity of hydrolysates, isolated peptides or any other antimicrobial agents. Each well contained 100 μL of Mueller–Hinton medium and was supplemented with 100 μL of peptide fraction (80 mg/mL). In total, 100 μL of the tested strain pre-diluted in Mueller–Hinton to a final bacterial load of 10^5^ CFU/mL was added last in each well. The inhibition of bacterial growth was monitored by measuring absorbance at 600 nm on a microplate reader (Safas, model MP96 UV-Vis Spectrophotometer, Agilent Technologies, Santa Clara, CA, USA) after 24 h of incubation at 37 °C. All measurements of MIC values were repeated in triplicate. The absorbance values of wells containing peptide hydrolysates were compared to those of negative controls consisting of a Mueller–Hinton medium and control culture. All the data are expressed as mean ± SD and are the mean of three replicates.

##### Antifungal Activity

Fungal strains used in this study were chosen because they represent some of the most abundant fungal species causing significant food contamination; they were isolated from the environment, food or dairy products [37]. Five fungal strains were used for determination of antifungal activity: *Aspergillus Niger* (3071-13), *Mucor racemosus* (LMA-722), *Penicillium crustosum* (27,159), *Rhodotorula mucilaginosa* (27,173) and *Paecilomyces spp* (5332-9a). These microorganisms were maintained in glycerol stocks at −80 °C. Then they were recovered on Potato Dextrose Agar (PDA, BD-Difco, Sparks, MD, USA) plates and incubated at 25 °C for a week for the molds and for 72 h for *Rhodotorula mucilaginosa*. For *Rhodotorula mucilaginosa,* cells were then recovered with a swab and incubated on PDA plates for 48 h at 25 °C. For the four molds, conidia were recovered with a swab soaked with peptone water containing 10% Tween 80 and were inoculated on PDA plates for another week at 25 °C. Conidia and vegetative cells of *Rhodotorula mucilaginosa* were then harvested using the same method and transferred in peptone water. Cell concentration was measured by an automated cell counter (Invitrogen™ AMQAF1000, Oakville, ON, Canada) and adjusted to ×10^6^ CFU/mL after dilutions in peptone water to obtain the same inoculum concentration for all the strains. The antifungal activity of extracts was assessed in agar diffusion assays. Briefly, a volume of 250 µL of each antifungal culture was then incorporated into falcon tubes containing 25 mL of PDA medium at 45 °C to obtain a final inoculum concentration of ×10^4^ CFU/mL. Inoculated media were then poured into sterile Petri dishes of 100 mm in diameter and solidified. Eights wells were uniformly dug on each inoculated PDA plate using a 5 mL serological pipette. In each well, 80 μL of hemoglobin solution at 10 and 20 mg/mL were spilled. The agar plates were incubated at 25 °C for 48 h, then the diameter of the inhibition zones indicating antifungal activity was measured (in millimeter), as indicated by the Clinical and Laboratory Standards Institute (CLSI 2016, CLSI 2017). A solution of 16.7 µg/mL natamycin (Sigma-Aldrich, Saint-Quentin Fallavier, France) was used as a positive control and sterile distilled water as negative control. All the data are expressed as mean ± SD and are the mean of three replicates.

##### Antioxidant Bioactivities

Four chemical in vitro assays, based on different antioxidant mechanisms, were used in this study to evaluate the antioxidant activity of our generated peptides.

- Antioxidant assay using the β-carotene bleaching method

The ability of the hydrolysate to prevent the bleaching of β-carotene was determined as described by Koleva et al. [38]. Briefly, a stock solution of β-carotene (4 mg), linoleic acid (100 μL) and Tween-40 (800 μL) was dissolved in 4 mL of chloroform. The latter was completely evaporated under vacuum (rotary evaporator, Heidolph, Schwabach, Germany) at 45 °C, then 400 mL of distilled water was added, and the resulting mixture was vigorously stirred. The β-carotene/linoleic acid emulsion obtained was freshly prepared before each experiment. An aliquot (5 mL) of the emulsion was transferred to tubes containing 500 μL of each sample prepared in ultrapure water at different concentrations. The absorbance at 470 nm (Shimadzu UV-1650 PC Spectrophotometer, shimadzu corporation, Kyoto, Japan) of each sample was measured before and after incubation at 50 °C for 2 h. Butylated hydroxytoluene (BHT) at a concentration of 0.5 mg/mL was used as positive control. Relative antioxidant activity was calculated according to Equation (1):

(1) $$\mathrm{RAA}\left(\%\right)=\;\frac{A\;\mathrm{sample}}{A\;\mathrm{control}}\;\times \;100$$

where A blank consisted of 500 μL of distilled water instead of the sample solution, A sample is the absorbance of samples (with the emulsion) and A control is the absorbance of BHT (with the emulsion). All the data are expressed as mean ± SD and are the mean of three replicates.

- DPPH radical scavenging capacity

The DPPH (1,1-diphenyl-2-picrylhydrazyl) radical scavenging capacity of samples was determined as described by Bersuder et al. [39]. A volume of 500 μL of each sample prepared in ultrapure water at different concentrations was added to 250 μL of 99% ethanol and 375 μL of DPPH solution (0.02% in ethanol) as free radical source. The mixtures were shaken and then incubated for 60 min in a dark room at 30 °C. Scavenging capacity was measured spectrophotometrically (Shimadzu UV-1650 PC Spectrophotometer, shimadzu corporation, Kyoto, Japan) by monitoring the decrease in absorbance at 517 nm. Lower absorbance of the reaction mixture indicated higher DPPH free radical scavenging activity. BHT was used as positive control. DPPH radical scavenging capacity was calculated according to Equation (2):

(2) $$\mathrm{DPPH}\;\mathrm{radical}\;\mathrm{scavenging}\;\mathrm{activity}\;(\%)\;=\;\frac{A\;\mathrm{blank}-A\;\mathrm{sample}}{A\;\mathrm{control}}\times 100$$

where A blank is the absorbance of the control reaction (containing all reagents except the sample), A sample is the absorbance of samples (with the DPPH solution) and A control is the absorbance of BHT (with the DPPH solution). DPPH^+^ radical scavenging activity was expressed as the half-maximal inhibition concentrations (IC_50_) and Trolox Equivalent Antioxidant Capacity (TEAC) values. The IC_50_ was calculated graphically by linear regressions of the percentage curves inhibition according to different concentrations of the hydrolysates tested. It represents the value of the sample concentration required to inhibit 50% of DPPH radicals. The lower this value, the more the compound was anti-free radical [40]. The TEAC value was defined as the concentration of standard Trolox, a water-soluble vitamin E analogue that exhibited the same antioxidant capacity as a 1 mg/mL solution concentration of the antioxidant compound under investigation [41]. It is used as a reference antioxidant in biology for evaluation of resistance to oxidative stress. All analyses were carried out in triplicate and results represented the mean values with standard deviations.

- Antioxidant properties products by ABTS assay

The ABTS (2,2′-azino-bis 3-ethylbenzothiazoline-6-sulphonic acid) radical scavenging activities were determined according to the method described by Re et al. [42]. The cationic radical ABTS^+^ was obtained by aqueous reaction between 7 mM ABTS and 4.95 mM potassium persulfate. This was prepared 12 to 16 h before use. The solution was stable when stored at room temperature and protected from light. A part of this solution was diluted with ethanol at 30 °C, in order to obtain an absorbance at 734 nm of less than 1. The hydrolysates were previously dissolved in ultrapure water at different concentrations and then, 10 μL of each sample were mixed with 1 mL of the diluted ABTS^+^ solution. The absorbance of ABTS^+^ was measured at 734 nm (Shimadzu UV-1650 PC Spectrophotometer, shimadzu corporation, Kyoto, Japan) and at 30 °C exactly 6 min after the initial mixing. Appropriate solvent blanks were run in each assay. The results are expressed as the percentage inhibition according to the following Equation (3):

(3) $${\mathrm{ABTS}}^{+}\;\mathrm{radical}\;\mathrm{scavenging}\;\mathrm{activity}\;(\%)\;=\;(1\;-\;\frac{A\;\mathrm{sample}}{A\;\mathrm{Blank}})\times 100$$

where A blank is the absorbance of the control reaction (containing all reagents except the sample) and A sample is the absorbance of samples (with the ABTS solution). ABTS^+^ radical scavenging activity was expressed as the half-maximal inhibition concentration (IC_50_) and TEAC values. All the data are expressed as mean ± SD and are the mean of three replicates.

- Evaluation of total antioxidant capacity

Total antioxidant capacity of the hydrolysates was determined according to the method described by Prieto et al. [43]. It is a quick and simple method for determining antioxidant activity, based on an oxidation–reduction reaction. This technique is based on the reduction of molybdenum Mo present as molybdate MoO_4_^2−^ ions to molybdenum MoO^2+^ in the presence of the hydrolysate to form a phosphate/molybdenum (Mo_3_O_16_P_4_) green complex at acidic pH. This test allowed us to evaluate the antioxidant status of the peptides present in the hydrolysates as reducing agents in a colorimetric redox reaction. Peptide hydrolysates were previously dissolved in ultrapure water at different concentrations (2.5 mg/mL, 5 mg/mL, 10 mg/mL and 20 mg/mL) and then an aliquot of 300 μL of each sample was mixed with 3 mL reagent (0.6 M sulfuric acid, 28 mM sodium phosphate, and 4 mM ammonium molybdate). The tubes were capped and incubated in a thermal block at 95 °C for 90 min. The absorbance of the aqueous solution of each sample was measured at 695 nm (Shimadzu UV-1650 PC Spectrophotometer, shimadzu corporation, Kyoto, Japan), after cooling at room temperature, against a blank. A typical blank solution contained 3 mL of reagent solution and the appropriate volume of the same solvent used for the sample, and it was incubated under the same conditions as the rest of the samples. BHT (0.5 mg/mL) was used as positive standard. A Trolox solution at different concentrations, ranging from 0 to 0.75 mg/mL, was used for calibration. The antioxidant activity is expressed in Trolox Equivalent Antioxidant Capacity (TEAC) values (mg/mL) using the Equation (4) given by the calibration line:

A = a × [C_Trolox_] + b (4)

where A the absorbance at 695 nm, C the equivalent antioxidant concentration (mg/mL). The concentration is determined according to the equation of the standard range curve of a reference antioxidant such as: Trolox, a and b, respectively, the origin and the slope of the Trolox calibration line. All the data are expressed as mean ± SD and are the mean of three replicates.

#### 2.4.3. Statistical Analyses

All analyses were performed in triplicate and three independent repetitions were done for each condition. Data were subjected to one-way or two-way analyses of variance (ANOVA). Tukey tests were also performed on data using SigmaPlot software (Version 14.0) to determine which treatment was statistically different from the others at a probability level *p* of 0.05.

## 3. Results and Discussion

### 3.1. Identification and Characterization of the Peptide Population Derived from EDBM-MCP and EDBM-AEM

In our study, «Zipper» mechanism allowed obtaining a large peptide population in the three conditions as described by Abou-Diab et al., 2020 (part 1) [26]. However, mass spectrometry is required to identify these peptide populations and to be able to compare them with control.

#### 3.1.1. Schematic Representations

For readability, the results will be described in the form of a figures presenting the amino acid sequence of hemoglobin chains and all of the peptides identified for the comparison between the three pepsic hydrolysates derived from the conventional hydrolysis process, EDBM-MCP and EDBM-AEM, respectively. Peptide sequences from the alpha and beta-chains of hemoglobin, found by mass spectrometry analysis, in the protein hydrolysate of the conventional hydrolysis process, EDBM-MCP and EDBM-AEM are shown, respectively, in supplementary material Figures S1–S6. The recovery rates for the alpha chain were 98.5% (112 peptides), 98.5% (123 peptides) and 91.5% (110 peptides) for conventional hydrolysis process, EDBM-MCP and EDBM-AEM, respectively, while for the beta chain, the respective recovery rates were 100% (106 peptides), 100% (110 peptides) and 98% (106 peptides). The majority of the sequences of alpha chain were found for control and EDBM-MCP among the peptides identified by Peaks Studio, while for EDBM-AEM some short sequences were not found, but the numbers of sequences identified were still very high (more than 91%). Moreover, the majority of the sequences of beta chains were found for control, EDBM-MCP and EDBM-AEM. We noticed that in these three conditions more than 91% of α and β chain were recovered, this showed the great resemblance between the peptides produced even with the new process which did not use chemical solvents.

#### 3.1.2. Venn Diagrams

**Figure 2:**
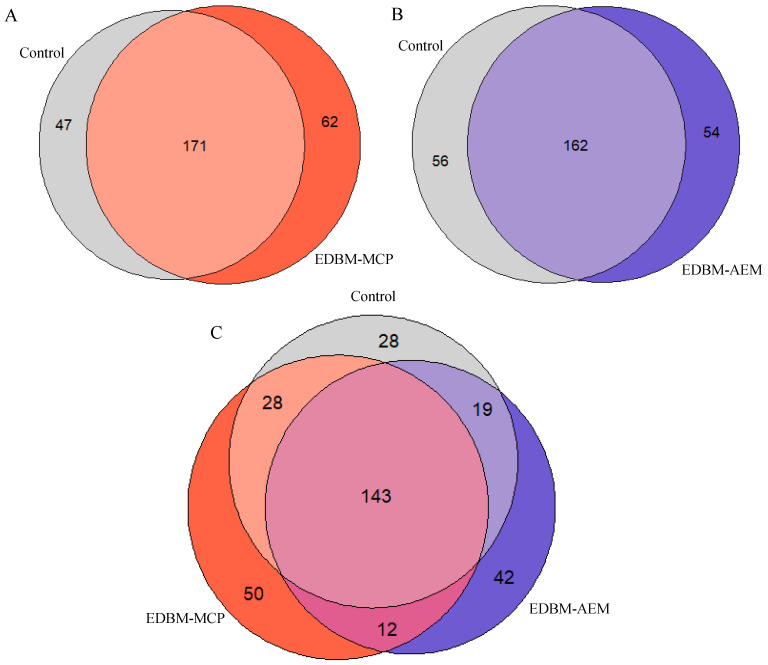
Venn diagram showing the different peptide populations produced by control and electrodialysis with bipolar membrane (EDBM)-MCP (**A**), control and EDBM-AEM (**B**) and control, EDBM-MCP and EDBM-AEM (**C**).

Venn diagrams showed all possible peptide population relations between control, EDBM-MCP and EDBM-AEM (Figure 2A–C). As can be seen, Figure 2A showed 171 peptides in common between control and EDBM-MCP. Figure 2B showed 162 peptides in common between control and EDBM-AEM. Moreover, the Venn diagram in Figure 2C showed 143 peptides in common between control, EDBM-MCP and EDBM-AEM. By using bipolar membrane electrodialysis with the ion-exchange membranes, whether cationic or anionic, we obtained roughly the same result. The bipolar membrane made it possible to reproduce the same peptide population as in the control under normal conditions with a slight difference because the three conditions do not follow the same parameters throughout the process as shown equally by Abou-Diab et al. (part 1) [26].

### 3.2. Identification of Bioactive Peptides Resulting from Bovine Hemoglobin Hydrolysis by EDBM-MCP and EDBM-AEM

One of the most important aim of this study was to identify the bioactive peptides obtained following the hydrolysis of bovine hemoglobin in EDBM and to compare them with the peptides obtained in control. Bioactive peptide sequences identified by UPLC-MS/MS are presented in Table 1. 

Among this large peptide population, numerous bioactive peptides were found in EDBM-MCP and EDBM-AEM, after three hours of hydrolysis, using bipolar membranes and without the use of chemical acids. The peptides identified by UPLC-MS/MS and compared with the literature show several bioactivities: antimicrobial [6,8,44], Hematopoietic [12], opioid [9,10,11], potentiator of bradykinin [9,33], Coronaro-constrictor [45], antihypertensive [13] and antioxidant [17]. The majority of the identified bioactive peptides were the same under all three conditions. The three hydrolysis processes produced 11 peptides in common having antimicrobial activity, 5 opioid peptides, 2 potentiator of bradykinin, 1 antihypertensive and 1 antioxidant peptide in common. The α33-46 which have an antimicrobial activity appeared in the EDBM-MCP and did not appear in the other conditions. Furthermore, α76-82 and β32-36 peptides which have hematopoietic and Coronaro-constrictor activity, respectively, appeared in the EDBM-MCP and did not appear in the other conditions. The bioactive peptides (α33-46, α76-82 and β32-36) presented in the EDBM-MCP and not present in the conventional and EDBM-AEM hydrolysis were precursors. Being an intermediate peptide, α33-46, α76-82 and β32-36, were rapidly hydrolyzed and no longer present after 3 h of hydrolysis in control and EDBM-AEM which agreed with the work of Dubois et al. [44], for bovine hemoglobin. Their presence in the EDBM-MCP and their absence in the conventional and EDBM-AEM conditions means that there was a slow-down in enzymatic kinetics during the hydrolysis of hemoglobin in EDBM-MCP as proposed by Abou-Diab et al. [26] which confirms the result found above in Venn diagrams.

### 3.3. Biological Activities of the Hydrolysates Obtained from EDBM-MCP and EDBM-AEM

#### 3.3.1. Antimicrobial Test

##### Antibacterial Activity

The results presented in Table 2 showed that, at 20 mg/mL, all hemoglobin hydrolysates derived from the three hydrolysis processes displayed antibacterial activity against the six tested bacterial species, with a clear growth inhibition zone. There is no significant difference in antibacterial activity between the hydrolysates derived from different conditions against a tested strain. Among the six bacterial strains, *S. aureus* and *K. rhizophila* presented the highest sensitivity to the hydrolysates. The hydrolysates of all conditions were noted to have strong protective effect against pathogenic and non-pathogenic bacteria.

##### MIC Determination of Antibacterial Peptide Hydrolysates

The hydrolysates were assessed for their MIC values in mg/mL because they contained multiple peptides. All samples showed activity against Gram-positive and Gram-negative bacteria. The results of MIC confirmed the findings previously found with the Agar Diffusion Method. However, the MIC values recorded for the active peptides against *S. aureus, L. monocytogenes* and *K. rhizophila* (between 0.31 to 2.5 mg/mL) were considerably lower than the MIC values registered against *M. luteus, E. coli and S. Newport* (between 5 to 10 mg/mL).

The antimicrobial effect of control and EDBM-AEM hydrolysate corresponded to MIC values of 0.31 mg/mL for *S. aureus* and 0.62 mg/mL for EDBM-MCP hydrolysates, which were statistically different (*p* < 0.05). Control hydrolysate displayed important antibacterial activity against *L. monocytogenes*, with MIC value of 1.25 mg/mL lower than the MIC value of EDBM-MCP and EDBM-AEM hydrolysates which have a value of 2.5 mg/mL. The hydrolysate from all conditions also showed antibacterial activity against *K.* rhizophila with identical MIC values of 0.31 mg/mL. The MIC value of 5 mg/mL for *M. luteus* obtained for control hydrolysate was statistically different (*p* < 0.05) from MIC values of 10 mg/mL for EDBM-MCP and EDBM-AEM hydrolysates. Identical antibacterial activities were also observed for all hydrolysates against *E. coli* and *S. Newport* with a MIC value of 10 mg/mL (Table 3).

In this study, we demonstrated that bovine hemoglobin hydrolysates, derived from conventional hydrolysis or from hydrolysis in EDBM, possessed antimicrobial activities against microorganisms that are of interest in the food industry. Our findings are in line with previous works reported by Zouari et al. [46] who showed the inhibitory effect of bovine or porcine hemoglobin hydrolysates against *M. luteus, L. Innocua, E. coli* and *S. Newport*. The hemoglobin peptides could be candidate for food preservation. In poultry feeding, these results are promising for the development of an alternative or supplement for reducing the use of antibiotics [47].

#### 3.3.2. Antifungal Test

**Figure 3:**
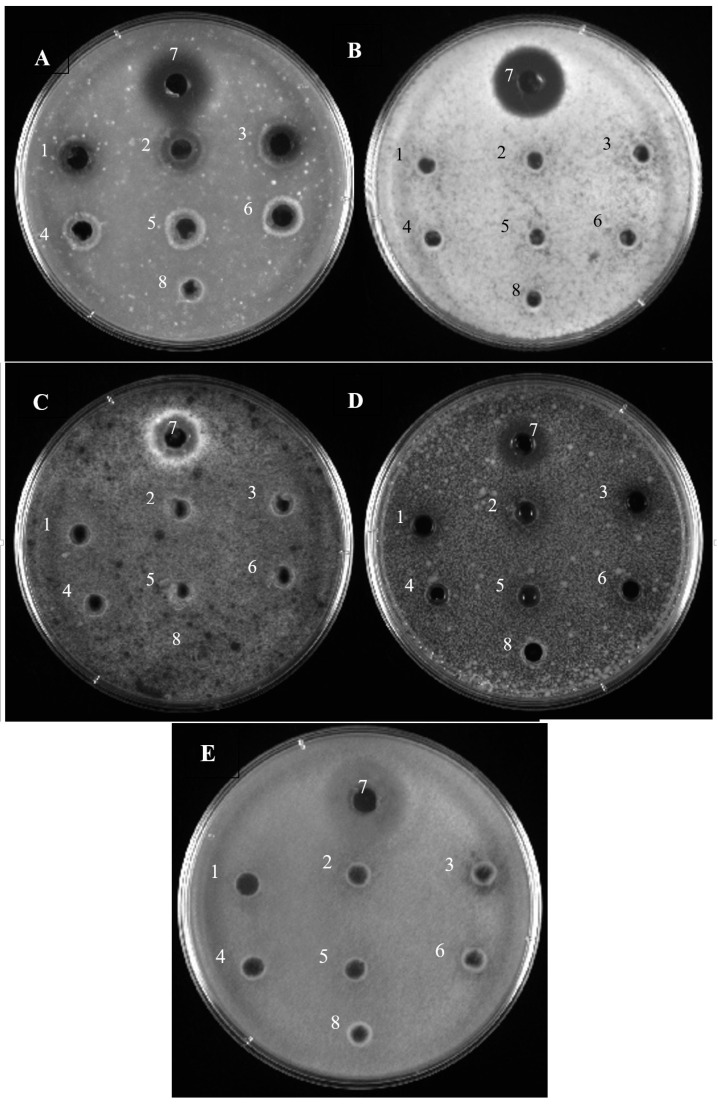
Antifungal activity of bovine hemoglobin hydrolysates produced by three different hydrolysis processes on Potato Dextrose Agar (PDA) overlaid with a suspension (104 spores per plate) of Paecilomyces spp (**A**), Penicillium crustosum (**B**), Aspergillus Niger (**C**), Rhodotorula mucilaginosa (**D**) and Mucor racemosus (**E**), 1–8 refer respectively to EDBM-MCP (20 mg/mL), EDBM-AEM (20 mg/mL), Hydrolysis Control (20 mg/mL), EDBM-MCP (10 mg/mL), EDBM-AEM (10 mg/mL), Hydrolysis Control (10 mg/mL), control +, control −.

Antifungal activities of bovine hemoglobin hydrolysates (10 and 20 mg/mL) obtained with treatment by pepsin are shown in Figure 3. Protein hydrolysates from hydrolysis control, EDBM-MCP and EDBM-AEM at a concentration of 20 mg/mL were strongly active against *Paecilomyces spp*, inducing zones of inhibition of 12.70 mm while hydrolysates at a concentration of 10 mg/mL induced a zone of inhibition of 11.90 mm, statistical analysis demonstrated that there is significant difference (*p* < 0.05) between these two values. The control hydrolysate at a concentration of 20 mg/mL was more active against *Penicillium crustosum* (11.90 mm inhibition zone) than with EDBM-MCP and EDBM-AEM hydrolysates at the same concentration (9.52 mm inhibition zone). All hydrolysates at a concentration of 10 mg/mL and 20 mg/mL were active against *Aspergillus Niger* producing a 4.76 mm inhibition zone in comparison with negative control, which has no inhibition zone. Statistical analysis demonstrated that there is significant difference (*p* < 0.05) in the inhibition zones formed by the hydrolysates against *Rhodotorula mucilaginosa*. At 20 mg/mL, the control hydrolysate presented an inhibition zone (11.11 mm) larger than the one of EDBM-AEM hydrolysate (9.52 mm) and even larger than by EDBM-MCP (4.76 mm). Always at 20 mg/mL, the control hydrolysate presented a clear activity against *Mucor racemosus* (inhibition zone of 11.90 mm) statistically similar (*p* > 0.05) to EDBM-AEM (11.11 mm) but statistically higher (*p* < 0.05) than the one of EDBM-MCP (4.76 mm). At a concentration of 10 mg/mL, all hydrolysates were active against *Mucor racemosus* inducing similar inhibition zone of 4.36 mm (*p* > 0.05). These tests showed that the most effective strains (based on inhibition zone diameter) belonged to *Paecilomyces spp*, *Penicillium crustosum* and *Mucor racemosus*. It should be noted that hydrolysates with a higher peptide concentration show higher antifungal activity. The hydrolysates derived from EDBM have an inhibitory effect on the fungal growth of these strains despite the small differences observed with the control. By way of comparison, the antifungal activity of the goat milk whey hydrolysates at a final concentration of 250 mg/mL induced inhibition zone of 8 mm against *Penicillium camemberti* and <8 mm against *Penicillium roqueforti* while the antifungal activity of bovine hemoglobin hydrolystes (20 mg/mL) derived from control, EDBM-MCP and EDBM-AEM induced inhibition zone >8 mm against *Penicillium crustosum* [48]. In this study, EDBM is an appealing technology allowing to produce hydrolysates with an interesting antifungal activity. These results are promising for the development of an alternative or supplement for reducing the use of antifungal chemical additives and drugs.

To the best of our knowledge, this was the first study demonstrating the ability of bioactive peptides derived from the hydrolysis of bovine hemoglobin to inhibit fungal growth.

#### 3.3.3. Antioxidant Activities of Bovine Hemoglobin Hydrolysates

- β-carotene bleaching inhibition activity

Lipid oxidation products react with proteins causing their oxidation. Carbohydrates are also susceptible to oxidation, but they are less sensitive than lipids and proteins [49]. In this study, lipid peroxidation inhibition activity of conventional hydrolysis (control), EDBM-MCP and EDBM-AEM hydrolysates was determined by assessing their ability to inhibit oxidation of linoleic acid in an emulsified model system.

**Figure 4:**
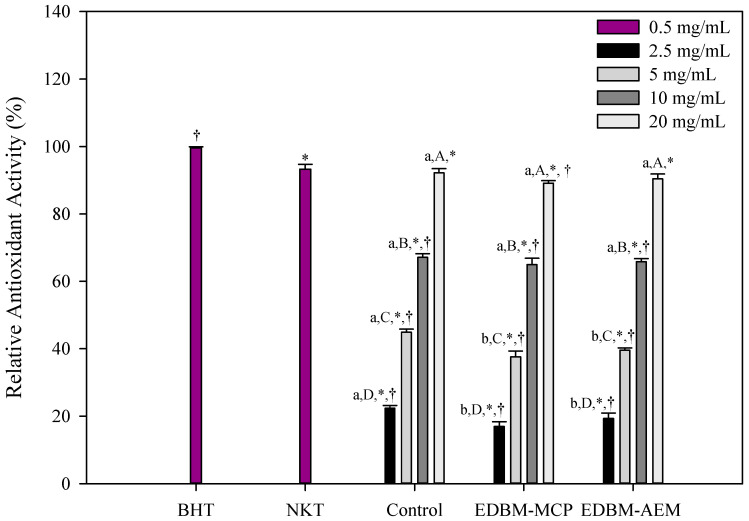
β-carotene bleaching inhibition activity of hemoglobin hydrolysates at different concentrations in Control, EDBM-MCP and EDBM-AEM. Values with (*) symbol are significantly different from BHT; Values with (†) symbol are significantly different from neokyotorphin (NKT); values with different lowercase letter (a-b) within the same concentration are significantly different; values with different capital letter (A-B-C-D) within the same condition are significantly different (*p* < 0.05, Tukey).

The antioxidative activity of control, EDBM-MCP and EDBM-AEM hydrolysates measured by the β-carotene bleaching assay are represented in Figure 4. Different concentrations of hydrolysates were tested and compared to the standard antioxidant, BHT (0.5 mg/mL) and synthetic bioactive peptide derived from hemoglobin hydrolysis, neokyotorphin (NKT, 0.5 mg/mL). All hydrolysates inhibited the oxidation of β-carotene at different degrees according to the concentration. The more the value is close to 100%, the more the antioxidant activity of the tested product corresponds to that of the BHT reference antioxidant (99.56 ± 0.36%). The relative antioxidant activity (RAA) of NKT is 93.26 ± 1.4%, statistically lower (*p* < 0.05) but close to the one of BHT. This peptide, therefore, has a lipid protection function close to the one found for BHT. At a concentration of 10 mg/mL and 20 mg/mL, there was no significant difference (*p* > 0.05) in antioxidant activity between control, EDBM-MCP and EDBM-AEM. However, at a concentration of 2.5 mg/mL and 5 mg/mL, there was a significant difference (*p* < 0.05) in antioxidant activity between EDBM-MCP and EDBM-AEM. Furthermore, a concentration of 20 mg/mL showed a significantly (*p* < 0.05) higher antioxidant activity (92.15 ± 1.29%; 89.1 ± 0.78%; 90.38 ± 1.46%, respectively) than the other concentrations. Relative antioxidant activity (%) increased with increasing protein hydrolysates concentrations. By way of comparison, the relative antioxidant activity of phenolic compounds of olive oil extracted in a traditional way is 67.40 ± 1.02% compared to BHT at 0.5 mg/mL [50]. This equals to our hydrolysates dissolved at 10 mg/mL.

The concentration of 20 mg/mL of the hydrolysates and 0.5 mg/mL of NKT have antiradical activity close to that of BHT at 0.5 mg/mL. Bleaching of β-carotene has been slowed down significantly in both cases. These products can, therefore, be described as primary antioxidant scavengers of free radicals in emulsion, so they would allow lipid protection.

- DPPH free radical scavenging capacity

The DPPH radical scavenging activity method is based on the ability of trapping the DPPH radicals. The drop-in absorption followed by spectrophotometry makes it possible to measure the antioxidant activity.

**Figure 5:**
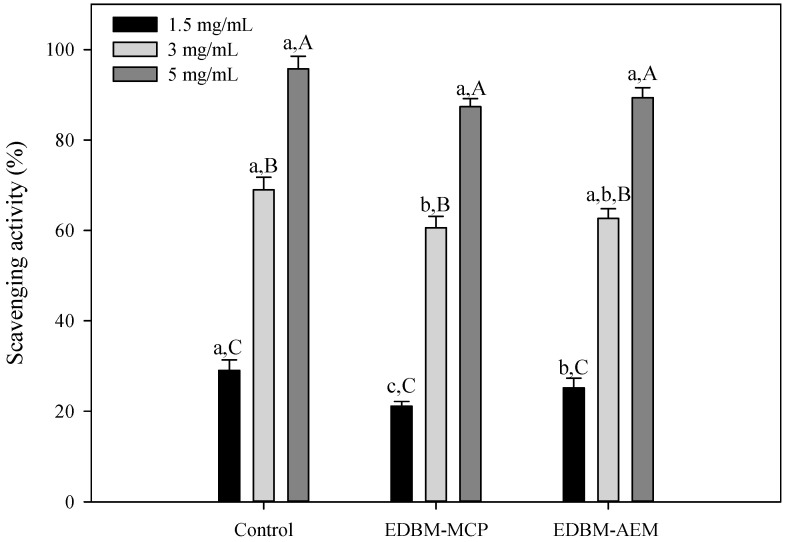
DPPH scavenging activities of hemoglobin hydrolysates in control, EDBM-MCP and EDBM-AEM at different concentrations. Values with different lowercase letter (a-b-c) within the same concentration are significantly different; values with different capital letter (A-B-C) within the same condition are significantly different (*p* < 0.05, Tukey).

The results (Figure 5) clearly indicated that hydrolysates obtained by treatment with pepsin exhibited a strong DPPH free radical scavenging activity. At 5 mg/mL, there was a significant difference (*p* < 0.05) in the antioxidant activity between control and EDBM-MCP while there was not significant difference (*p* > 0.05) between control and EDBM-AEM and between EDBM-AEM and EDBM-AEM. A concentration of 5 mg/mL for control, EDBM-MCP and EDBM-AEM showed significantly (*p* < 0.05) higher antioxidant activity (95.7 ± 2.8%; 87.3 ± 2.3%; 89.38 ± 2.19%, respectively) than the other concentrations. The antioxidant activity of bovine hemoglobin hydrolysates increased with increasing peptide concentrations. Our findings are in line with previous works reported by He Tang et al. [51] who reported that the DPPH-scavenging activity increased with increasing buckwheat protein hydrolysates concentrations (0–1.0 mg/mL) using alcalase.

The DPPH radical scavenging activities of the peptide hydrolysates were also expressed as the half-maximal inhibition concentrations (IC_50_) and Trolox Equivalent Antioxidant Capacity (TEAC). The IC_50_ value is applied as an indication to evaluate the scavenging activity. The lower the IC_50_ value, the higher the free radical scavenging ability. The comparative IC_50_ and TEAC values of the tested hemoglobin hydrolysates were shown in Table 4. The DPPH radical scavenging activity of control showed different (*p* < 0.05) IC_50_ (2.55 ± 0.02 mg/mL) compared to EDBM-MCP (2.86 ± 0.03 mg/mL) and EDBM-AEM (2.72 ± 0.04 mg/mL). However, NKT showed an IC_50_ (0.61 ± 0.05 mg/mL) close to that of Trolox (0.38 ± 0.03 mg/mL) but both statistically lower than the ones of control, EDBM-MCP and EDBM-AEM. The TEAC of control showed different (*p* < 0.05) values compared to EDBM-MCP and EDBM-AEM. In comparison with Trolox, the DPPH radical scavenging activity of control, EDBM-MCP and EDBM-AEM showed lower radical scavenging activity (TEAC value lower than 1). Additionally, the DPPH radical scavenging activity of NKT showed higher radical scavenging activity (0.62 ± 0.09) than the peptide hydrolysates.

- Antioxidant properties products by ABTS assay

The ABTS radical scavenging activity method is based on the ability of molecules to quench the ABTS radical cation, in comparison with Trolox, which was used as a reference molecule. The comparative IC_50_ and TEAC values of the tested hydrolysates are shown in Table 5.

To inhibit 50% of ABTS radicals, concentrations of hemoglobin hydrolysates between 3.42 and 3.66 mg/mL were needed. Statistical analysis showed that there was significant difference (*p* < 0.05) of IC_50_ for hemoglobin hydrolysates between control, EDBM-MCP and EDBM-AEM, but the values were very close. A concentration of 0.53 mg/mL of NKT inhibits 50% of ABTS radicals; this value is very close to Trolox but is statistically different (*p* < 0.05). Concerning TEAC, the ABTS radical scavenging activity of hemoglobin hydrolysates derived from control, EDBM-MCP and EDBM-AEM exhibited lower radical scavenging activity than Trolox (TEAC value lower than 1) while NKT showed a very close radical scavenging activity to Trolox (0.92 ± 0.01).

This result showed that hemoglobin hydrolysates have the ability to act as an antioxidative compound but due to the large number of bioactive peptides and the competitiveness between them they showed lower capacity compared to Trolox the reference antioxidant. In comparison, NKT which is the reference bioactive peptide showed an antioxidant activity close to Trolox.

- Evaluation of total antioxidant capacity

The total antioxidant capacity of the hydrolysates was evaluated at four different concentrations (2.5 mg/mL, 5 mg/mL, 10 mg/mL and 20 mg/mL), and Trolox was used as a reference antioxidant. The total antioxidant capacity of the hydrolysates was expressed as TEAC (mg/mL).

**Figure 6:**
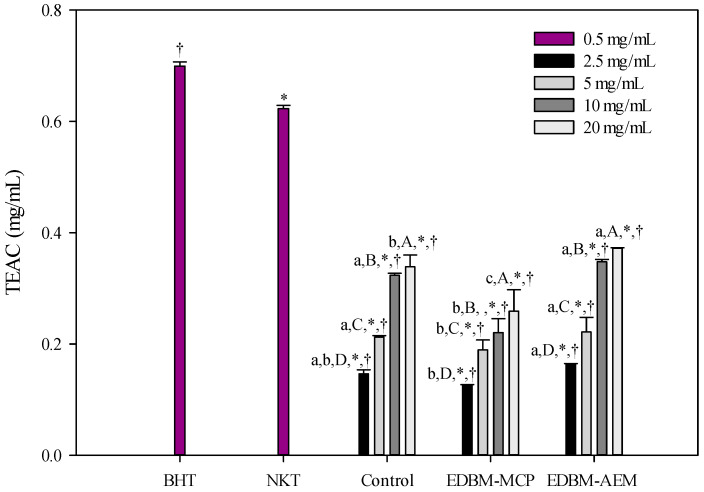
Total antioxidant capacity of hemoglobin hydrolysates in control, EDBM-MCP and EDBM-AEM at different concentrations. Values with (*) symbol are significantly different from BHT; Values with (†) symbol are significantly different from neokyotorphin; values with different lowercase letter (a-b) within the same concentration are significantly different; values with different capital letter (A-B-C-D) within the same condition are significantly different (*p* < 0.05, Tukey).

The results shown in Figure 6 clearly indicate a change in antioxidant capacity as a function of hydrolysate concentration. As expected, a concentration of 20 mg/mL for control, EDBM-MCP and EDBM-AEM showed significantly higher antioxidant activity than the other concentrations. EDBM-AEM (20 mg/mL) showed the highest TEAC compared to other conditions. There is significantly different antioxidant capacity between the different conditions for the same concentration; the TEAC increased from 0.146 ± 0.007, 0.126 ± 0.000 and 0.163 ± 0.001 mg/mL at a concentration of 2.5 mg/mL to 0.338 ± 0.021, 0.258 ± 0.038 and 0.371 ± 0.000 mg/mL at 20 mg/mL for conventional hydrolysis, EDBM-MCP and EDBM-AEM, respectively. BHT (0.5 mg/mL) has an antioxidant activity of 0.699 ± 0.007 mg/mL determined under the same conditions. TEAC of our 20 mg/mL hydrolysates is approximately two times lower than for 0.5 mg/mL BHT. The antioxidant activity of 20 mg/mL of hydrolysate is, therefore, equivalent to 0.25 mg/mL of BHT by the phosphor-molybdenum method.

Bovine hemoglobin hydrolysates of control, EDBM-MCP and EDBM-AEM have demonstrated important antioxidant properties and can be considered as promising antioxidants. The antioxidant properties of peptides are influenced by molecular size, composition, hydrophobicity and electron transferring ability of amino acid residues in the sequence [52]. Hydrolysis is required to generate smaller bioactive peptides that possess biological activity. Antioxidant activity can be due to the total denatured hemoglobin favoring the «Zipper» mechanism which made it possible to produce low molecular weight hydrolysates having important radical scavenging activities.

## 4. Conclusions

The electrodialysis process using bipolar membranes combined with other ion-exchange membranes has been successful, and it has made it possible to adjust the pH and make the hydrolysis under the right conditions using the H^+^ and OH^−^ generated by the bipolar membranes and without the use of chemical solvents. By the «Zipper» mechanism, EDBM, whatever the configuration of MCP or AEM, allowed for a recovery rate of the alpha and beta chain of bovine hemoglobin greater than 91% and similar to the conventional hydrolysis. Furthermore, following the identification of peptide sequences by mass spectrometry, EDBM was demonstrated to produce 17 bioactive peptides that are already known for their activities. More specifically the production of neokyotorphin (α137-141) which has shown several biological activities, which has a great importance in the food industry.

The results of the present work showed an excellent microbial growth inhibition of bovine hemoglobin peptide hydrolysates derived from conventional hydrolysis or from hydrolysis by EDBM against *S. aureus*, *L. monocytogenes*, *K. rhizophila*, *M. luteus*, *E. coli* and *S. Newport.* This study reports, for the first time, the capacity of these hydrolysates to inhibit the growth of *Aspergillus Niger*, *Mucor racemosus*, *Penicillium crustosum*, *Rhodotorula mucilaginosa* and *Paecilomyces spp*. Moreover, these results confirmed that bovine hemoglobin hydrolysates, whatever their mode of production, had antioxidant capacities. Hence, some peptides in the hydrolysates are new potent free radical scavengers that could be used as natural antioxidants in food matrices or products.

This new green application of EDBM and the production of antimicrobial, antifungal and antioxidant peptide, from waste, fits perfectly with the concept of circular economy. However, the issue of fouling highlighted and explained in the technological part of this study (Abou-Diab et al., 2020 (part 1) [26]) has to be fixed before further steps. Different approaches are already being considered and are under way, such as applying pulsed electric field, changing the EDBM configuration or modifying the ion-exchange membranes [26,53].

## Figures and Tables

**Table 1:** Bioactive peptide sequences identified by UPLC-MS/MS resulting from hydrolysis of bovine hemoglobin for the three different hydrolysis processes tested.

Biological Activity	Position	Sequence	Molecular Weight (Da)	Control	EDBM-MCP	EDBM-AEM
Antimicrobial	α1-29	VLSAADKGNVKAAWGKVGGHAAEYGAEAL	2839	+	+	+
	α1-27	VLSAADKGNVKAAWGKVGGHAAEYGAE	2655	+	+	+
	α1-23	VLSAADKGNVKAAWGKVGGHAAE	2235	+	+	+
	α34-46	LSFPTTKTYFPHF	1585	+	+	+
	α33-46	FLSFPTTKTYFPHF	1732	−	+	−
	α37-46	PTTKTYFPHF	1238	+	+	+
	α137-141	TSKYR	653	+	+	+
	β1-13	MLTAEEKAAVTAF	1381	+	+	+
	β126-145	QADFQKVVAGVANALAHRYH	2194	+	+	+
	β140-145	LAHRYH	795	+	+	+
	α99-105	KLLSHSL	796	+	+	+
	α99-106	KLLSHSLL	910	+	+	+
Hematopoietic	α76-82	LPGALSE	685	−	+	−
Opioid	α137-141	TSKYR	653	+	+	+
	β32-37	VVYPWT	763	+	+	+
	β31-37	LVVYPWT	876	+	+	+
	β32-40	VVYPWTQRF	1195	+	+	+
	β31-40	LVVYPWTQRF	1308	+	+	+
Potentiator of bradykinin	α129-134	LANVST	603	+	+	+
	α110-125	ASHLPSDFTPAVHASL	1649	+	+	+
Coronaro-constrictor	β32-36	VVYPW	663	−	+	−
Antihypertensive	α99-105	KLLSHSL	796	+	+	+
Antioxidant	α137-141	TSKYR	653	+	+	+

**Table 2:** Antibacterial activity of bovine hemoglobin hydrolysates obtained with treatment by pepsin.

Bacteria Strains	Bovine Hemoglobin Hydrolysates		
	Control	EDBM-MCP	EDBM-AEM
*Staphylococcus aureus*	+++	+++	+++
*Listeria monocytogenes*	++	++	++
*Micrococcus luteus*	+	+	+
*Kocuria rhizophila*	+++	+++	+++
*Escherichia coli*	+	+	+
*Salmonelle Newport*	+	+	+

Inhibition zones: +++: >1.5 cm; ++ 0.5–1.5 cm; +: <0.5 cm.

**Table 3:** MIC values of the antibacterial peptide hydrolysates.

Bacteria Strains	Bovine Hemoglobin Hydrolysates		
	MIC		
	Control	EDBM-MCP	EDBM-AEM
	mg/mL	mg/mL	mg/mL
*Staphylococcus aureus*	0.31 ± 0.0 ^a^	0.62 ± 0.0 ^b^	0.31 ± 0.0 ^a^
*Listeria monocytogenes*	1.25 ± 0.0 ^a^	2.5 ± 0.0 ^b^	2.5 ± 0.0 ^b^
*Micrococcus luteus*	5 ± 0.0 ^a^	10 ± 0.0 ^b^	10 ± 0.0 ^b^
*Kocuria rhizophila*	0.31 ± 0.0 ^a^	0.31 ± 0.0 ^a^	0.31 ± 0.0 ^a^
*Escherichia coli*	10 ± 0.0 ^a^	10 ± 0.0 ^a^	10 ± 0.0 ^a^
*Salmonelle Newport*	10 ± 0.0 ^a^	10 ± 0.0 ^a^	10 ± 0.0 ^a^

The minimum inhibitory concentration (MIC) of the peptide hydrolysates was determined in a microtiter plate assay system after 24 h of incubation at 30 or 37 °C. ^a,b^: Population means for each bacteria within each row with different letters are significantly different, similar letter means no significant difference *p* < 0.05 (Anova, Tukey).

**Table 4:** IC50 and Trolox Equivalent Antioxidant Capacity (TEAC) coefficients of hemoglobin hydrolysates for the DPPH method.

DPPH	Control	EDBM-MCP	EDBM-AEM	NKT	Trolox
IC_50_ (mg/mL)	2.55 ± 0.02 ^a^	2.86 ± 0.03 ^b^	2.72 ± 0.04 ^c^	0.61 ± 0.05 ^d^	0.38 ± 0.03 ^e^
TEAC	0.15 ± 0.03 ^a^	0.13 ± 0.02 ^b^	0.14 ± 0.01 ^c^	0.62 ± 0.09 ^d^	1 ^e^

^a–e^: Means values within each row with different letters are significantly different, similar letter means no significant difference at a probability level of 0.05 (Anova, Tukey).

**Table 5:** IC50 and TEAC coefficients of hemoglobin hydrolysates for ABTS method.

ABTS	Control	EDBM-MCP	EDBM-AEM	NKT	Trolox
IC_50_ (mg/mL)	3.42 ± 0.01 ^a^	3.66 ± 0.03 ^b^	3.55 ± 0.04 ^c^	0.53 ± 0.05 ^d^	0.49 ± 0.09 ^e^
TEAC	0.14 ± 0.03 ^a^	0.13 ± 0.02 ^b^	0.14 ± 0.01 ^a^	0.92 ± 0.01 ^c^	1 ^d^

^a–e^: Means values within each row with different letters are significantly different, similar letter means no significant difference *p* < 0.05 (Anova, Tukey).

## Supplementary Materials

The following are available online at https://www.mdpi.com/2077-0375/10/10/268/s1, Figures S1, S3 and S5: Schematic representation of the peptides resulting from the enzymatic hydrolysis of the α chain of bovine hemoglobin in control, EDBM-MCP and EDBM-AEM, respectively, Figures S2, S4 and S6: Schematic representation of the peptides resulting from the enzymatic hydrolysis of the β chain of bovine hemoglobin in control, EDBM-MCP and EDBM-AEM, respectively.

## Abbreviations

Hb	Hemoglobin
ED	Electrodialysis
AEM	Anion exchange membrane
MCP	Monovalent cation permselective membrane
BM	Bipolar membrane
