# Supplementary material for: Bovine Hemoglobin Enzymatic Hydrolysis by a New Eco-Efficient Process-Part II: Production of Bioactive Peptides
Source: Membranes (Basel). 2020 Sep 29;10(10):268. doi: 10.3390/membranes10100268 (PMC7600257; doi:10.3390/membranes10100268)
Supplement: Supplementary file 1 [file membranes-10-00268-s001.pdf]

Supplementary Materials: Bovine Hemoglobin Enzymatic Hydrolysis by a New Eco-Efficient Process-Part II: Production of Bioactive Peptides

Mira Abou-Diab [1,2,3,4], Jacinthe Thibodeau [1,2,3], Barbara Deracinois [4], Christophe Flahaut [4], Ismail Fliss [1,3], Pascal Dhulster [4], Laurent Bazinet [1,2,3,*,†] and Naima Nedjar [4,*,†]

1 Department of Food Science, Université Laval, Québec, QC G1V 0A6, Canada; mira.abou-diab.1@ulaval.ca (M.A.-D.); jacinthe.thibodeau.1@ulaval.ca (J.T.); ismail.fliss@fsaa.ulaval.ca (I.F.)
2 Laboratory of Food Processing and Electromembrane Process (LTAPEM), Université Laval, Québec, QC G1V 0A6, Canada
3 Institute of Nutrition and Functional Foods (INAF), Université Laval, Québec, QC G1V 0A6 Canada
4 UMR Transfrontalière BioEcoAgro N°1158, Université Lille, INRAE, Université Liège, UPJV, YNCREA, Université Artois, Université Littoral Côte d'Opale, ICV—Institut Charles Viollette, F-59000 Lille, France; barbara.deracinois@univ-lille.fr (B.D.); christophe.flahaut@univ-artois.fr (C.F.); pascal.dhulster@univ-lille.fr (P.D.)
* Correspondence: Laurent.Bazinet@fsaa.ulaval.ca (L.B.); naima.nedjar@univ-lille.fr (N.N.); Tel.: +1-418-656-2131 (ext. 407445) (L.B.); +33-3-2876-7390 (N.N.); Fax: +1-418-656-3353 (L.B.); +33-3-2876-7356 (N.N.)
† These authors contributed equally to this work

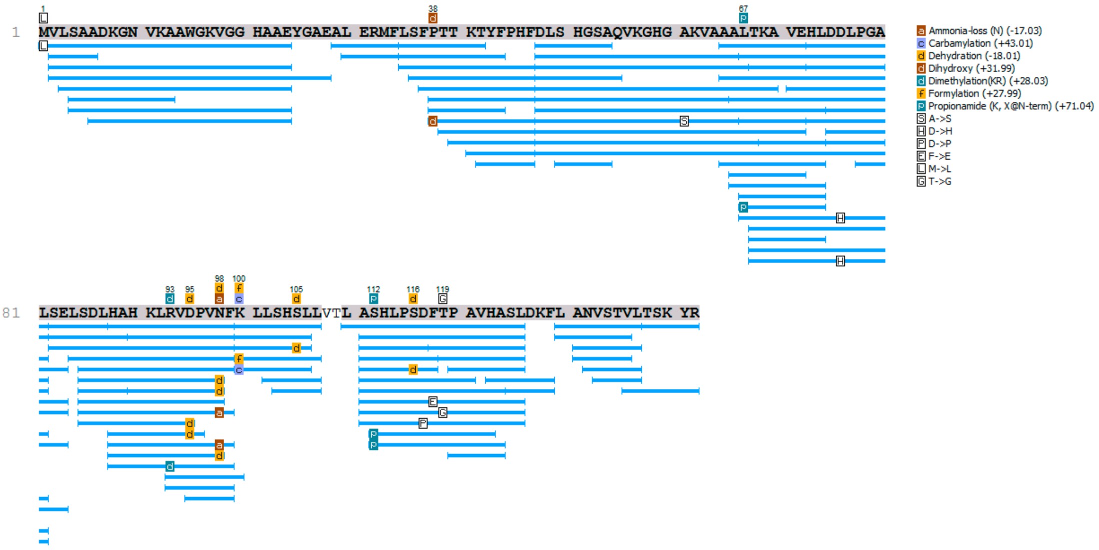

Figure S1. Schematic representation of the peptides resulting from the enzymatic hydrolysis of the α chain of bovine hemoglobin in control.

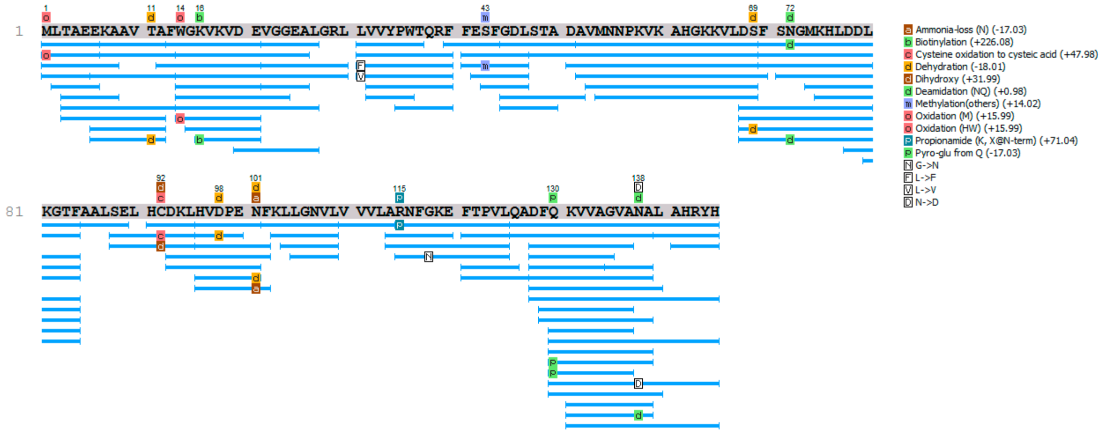

Figure S2. Schematic representation of the peptides resulting from the enzymatic hydrolysis of the β chain of bovine hemoglobin in control.

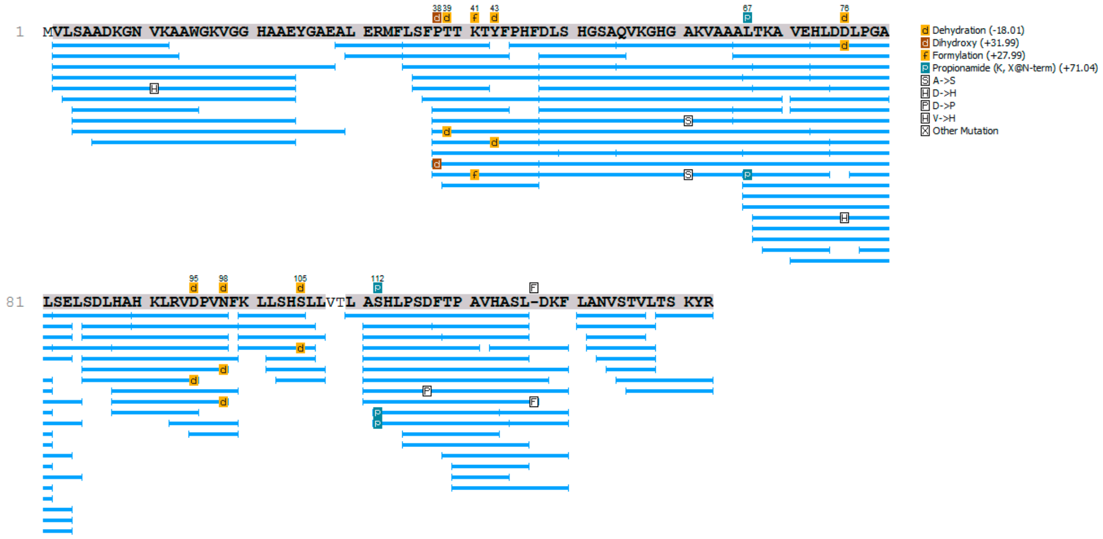

Figure S3. Schematic representation of the peptides resulting from the enzymatic hydrolysis of the α chain of bovine hemoglobin in EDBM-MCP.

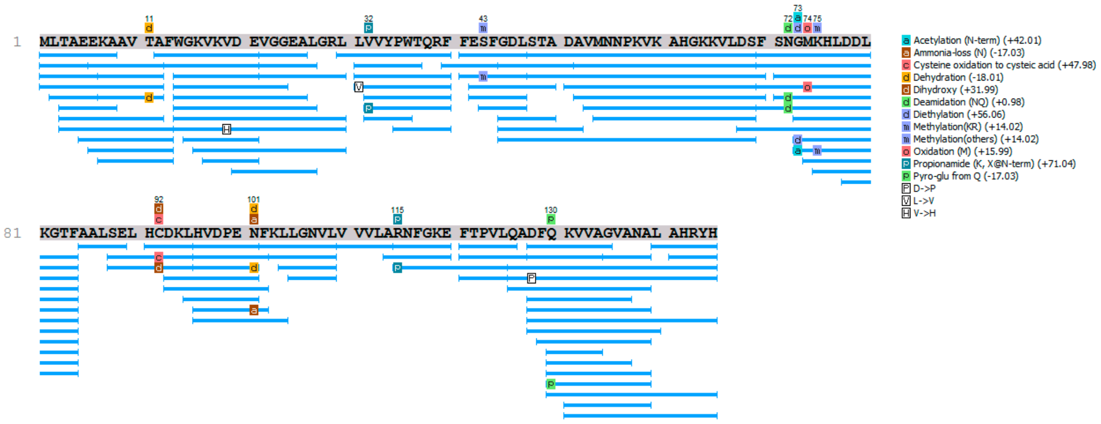

Figure S4. Schematic representation of the peptides resulting from the enzymatic hydrolysis of the β chain of bovine hemoglobin in EDBM-MCP.

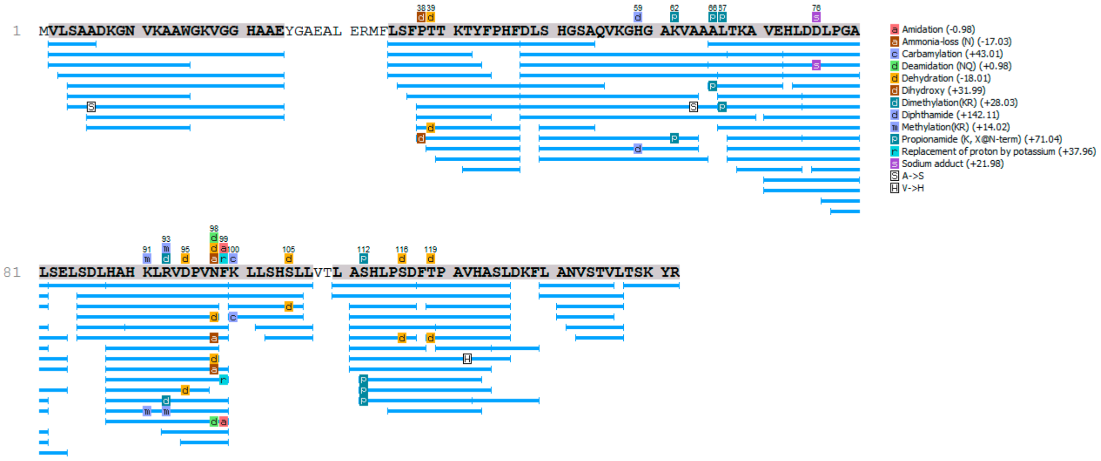

Figure S5. Schematic representation of the peptides resulting from the enzymatic hydrolysis of the α chain of bovine hemoglobin in EDBM-AEM.

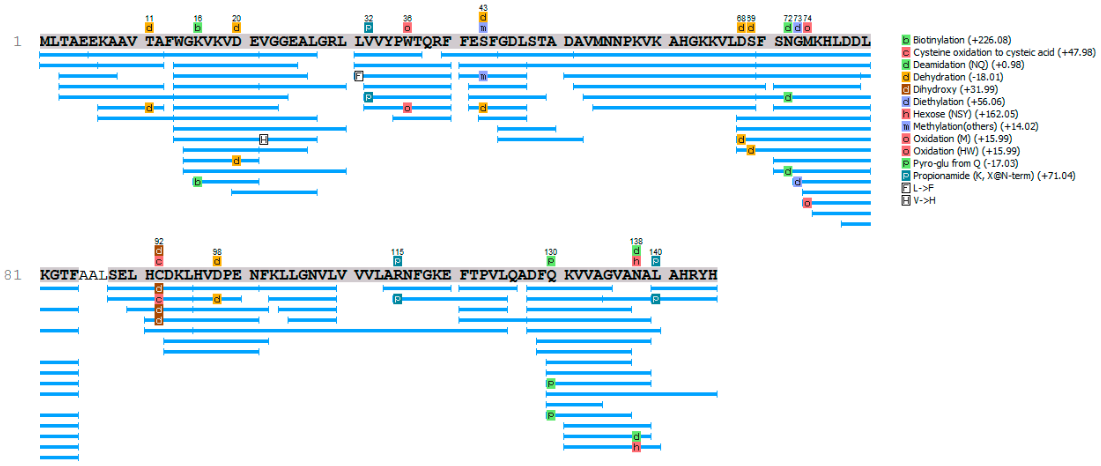

Figure S6. Schematic representation of the peptides resulting from the enzymatic hydrolysis of the β chain of bovine hemoglobin in EDBM-AEM.
